# Looking into a Conceptual Framework of ROS–miRNA–Atrial Fibrillation

**DOI:** 10.3390/ijms151221754

**Published:** 2014-11-26

**Authors:** Seahyoung Lee, Eunhyun Choi, Min-Ji Cha, Ki-Chul Hwang

**Affiliations:** 1 Institute for Bio-Medical Convergence, College of Medicine, Catholic Kwandong University, Gangneung-si, Gangwon-do 210-701, Korea; E-Mails: sam1017@ish.or.kr (S.-Y.L.); ehchoi@ish.or.kr (E.-H.C.); minjicha619@gmail.com (M.-J.C.); 2 Catholic Kwandong University International St. Mary’s Hospital, Incheon Metropolitan City 404-834, Korea

**Keywords:** atrial fibrillation (AF), reactive oxygen species (ROS), miRNA, arrhythmia

## Abstract

Atrial fibrillation (AF) has been recognized as a major cause of cardiovascular-related morbidity and mortality. MicroRNAs (miRNAs) represent recent additions to the collection of biomolecules involved in arrhythmogenesis. Reactive oxygen species (ROS) have been independently linked to both AF and miRNA regulation. However, no attempts have been made to investigate the possibility of a framework composed of ROS–miRNA–AF that is related to arrhythmia development. Therefore, this review was designed as an attempt to offer a new approach to understanding AF pathogenesis. The aim of this review was to find and to summarize possible connections that exist among AF, miRNAs and ROS to understand the interactions among the molecular entities underlying arrhythmia development in the hopes of finding unappreciated mechanisms of AF. These findings may lead us to innovative therapies for AF, which can be a life-threatening heart condition. A systemic literature review indicated that miRNAs associated with AF might be regulated by ROS, suggesting the possibility that miRNAs translate cellular stressors, such as ROS, into AF pathogenesis. Further studies with a more appropriate experimental design to either prove or disprove the existence of an ROS–miRNA–AF framework are strongly encouraged.

## 1. Introduction

Arrhythmia refers to any irregular rhythm of the heart, including both rhythms that are too fast (tachycardia) and rhythms that are too slow (bradycardia). Although most arrhythmias do not cause serious problems, atrial fibrillation (AF), the most common sustained type of arrhythmia, has been recognized as a major cause of cardiovascular-related morbidity and mortality [1,2]. Over the last decade, the mechanistic understanding of AF has advanced tremendously, and microRNAs (miRNAs) have become recent additions to the collection of biomolecules involved in AF development. In fact, the notion of miRNAs acting as AF modulators is not new; reviews regarding the role of miRNAs in AF have already been published [3,4]. However, to the best of our knowledge, no attempts have been made to investigate the possible involvement of reactive oxygen species (ROS), which have been independently linked to both AF [5] and miRNA expression in cardiovascular diseases [6], in the miRNA-mediated development of AF.

Therefore, in this review, we have attempted to find and to summarize possible connections that exist among AF, miRNAs and, particularly, ROS, to understand the interactions of the molecular entities underlying arrhythmia development in the hopes of finding unappreciated mechanisms of AF. These findings may lead us to innovative therapies for AF, which can be a life-threatening heart condition. This review represents an attempt to offer a new approach to understanding AF pathogenesis and focuses on the possible interplay between ROS and miRNA rather than on AF itself. Therefore, this review will not address the mechanical details of AF (for a comprehensive review on AF, see [4,7]), nor will it address the mechanical details of miRNAs or ROS. Rather, we will briefly discuss the key characteristics of AF and related molecules, because they are most likely the primary targets of ROS-mediated miRNA regulation in AF development. Additionally, the fundamentals of both miRNA and ROS will be discussed briefly, prior to investigating the possibility of ROS-dependent regulation of the miRNAs that have been linked to AF.

## 2. Atrial Fibrillation (AF) and Atrial Remodeling

“Atrial remodeling” is a key term in understanding the nature of AF and is defined as any persistent changes in atrial function and structure [8]. AF may be a final result of atrial remodeling induced by various pathologic conditions of the heart [9]; however, it may also be a major cause of remodeling, which perpetuates its progression. The two fundamental components of atrial remodeling are electrical remodeling, which occurs mainly due to alterations in ion channels [10,11], and structural remodeling, which may be caused by fibrosis [12]. Additionally, Ca^2+^ handling abnormalities [13] and autonomic nerve activation and remodeling [14] are also factors that may contribute to atrial remodeling.

### 2.1. Electrical Remodeling of the Heart

The electrical remodeling of cardiomyocytes involves the modulation of ions, especially Ca^2+^ and K^+^, and intercellular ion channels (*i.e.*, gap junctions), which participate in the electrical coupling of adjacent cells. Consequently, any ion channel, exchanger or pump, which is fundamentally a complex of proteins, may be subjected to miRNA-mediated regulation.

#### 2.1.1. Ca^2+^ Regulation and Key Molecules Involved

l-Type Ca^2+^ currents (*I*_CaL_) are decreased during AF [15]. l-Type Ca^2+^ channel (LTCC)-mediated Ca^2+^ influx into cardiomyocytes induces the secondary release of Ca^2+^ stored in the sarcoplasmic reticulum (SR) via ryanodine receptor 2 (RyR2) [16]. RyR2-mediated Ca^2+^ leakages have been linked to AF in animal models [17]. However, the re-uptake of cytosolic Ca^2+^ into the SR to maintain Ca^2+^ homeostasis is achieved via an SR Ca^2+^-adenosine triphosphatase (SERCA2a), which is under the inhibitory control of the SR-associated protein, phospholamban (PLB) [18]. Another ion channel responsible for the diastolic removal of the Ca^2+^ released during systole is the Na^+^/Ca^2+^ exchanger (NCX). The inward current carried by this exchanger is responsible for the delayed after depolarizations (DAD) observed in patients with chronic AF [19]. Additionally, the Ca^2+^/calmodulin kinase type 2 (CaMKII)-dependent phosphorylation of RyR2 has been linked to the depletion of SR Ca^2+^ storage and increased diastolic Ca^2+^ levels, which illustrates its involvement in arrhythmia development [20]. As for the relationship with ROS, the activities of some Ca^2+^ channels are known to be modulated by ROS. For example, it has been reported that ROS inhibit LTCC current [21] and SERCA2 activity [22], while increasing the activity of NCX [23] and RYR [24]. These results indicated that ROS might play an important role in atrial electrical remodeling, which underlies AF, by significantly altering Ca^2+^ handling by cardiomyocytes.

#### 2.1.2. K^+^ Regulation and Key Molecules Involved

K^+^ current plays a crucial role in the regulation of cardiomyocyte excitability; therefore, it has been implicated in AF development [25]. Encoded by the *KCNJ2* gene, the Kir2.1 proteins constitute the inward rectifier K^+^ channels found in cardiomyocytes [26]. Cardiac inward rectifier current (*I*_K1_), along with Kir2.1 expression, is reportedly increased during AF [27,28]. Another important channel controlling inward rectifier K^+^ current is the acetylcholine-activated potassium channel (*I*_KACh_ channel), which is composed of Kir3 subunits [29]. Activated by acetylcholine released from the vagus nerve, *I*_KACh_ facilitates strong inward rectifying K^+^ current that mediates the parasympathetic induction of bradycardia and subsequent decreases in cardiac contractility. Its importance in AF pathogenesis has been demonstrated using knockout animals [30].

Levels of the constitutive isoform, *I*_KAChc_, are increased in AF [31]. It has been reported that shortened action potential durations (APDs) are strongly associated with AF development [32], and *I*_KAChc_ is believed to be responsible for this phenomenon in tachycardia-remodeled atria, which contributes to contractile dysfunction in AF [33]. Additionally, the increased expression of small conductance Ca^2+^-activated K^+^ (SK) channels [34] and their contribution to AF maintenance by causing APD shortages have been described previously [35]. The activity of K^+^ channels can be modulated by ROS. For example, the Kv1.5 channel has been implicated in the development of AF [36], and blockers of the Kv1.5 channel have been investigated as therapeutic agents for AF [37,38]. According to a study examining the effects of H_2_O_2_ on this Kv1.5 channel, H_2_O_2_ treatment induced an increase in Kv1.5 current and accelerated the Kv1.5 channel [39], suggesting that such ROS-induced modulation of the K^+^ channel could contribute to the initiation of AF. Another example of ROS-induced modulation of the K^+^ channel was demonstrated in a study in which H_2_O_2_ inhibited the activity of the hERG1 K^+^ channel, resulting in APD prolongation [40].

#### 2.1.3. Gap Junction Ion Channels

In the heart, the electrical coupling of adjacent cardiomyocytes is mediated by gap junction ion channels consisting of connexins (Cx), such as Cx40 and Cx43. Cx40 is located primarily in atrial tissue and the conduction system, whereas Cx43 is the most abundant connexin isoform [41]. Gap junction ion channel malfunctions have been linked to AF-induced remodeling [42,43].

### 2.2. Structural Remodeling of the Heart

Structural remodeling refers to progressive morphological and functional alterations of atrial substrates, particularly the posterior wall of the left atrium. Various diseases and aging can induce these changes [44,45]. Hallmarks of structural remodeling include atrial dilation and progressive interstitial fibrosis. For example, heart failure-induced prominent ultra-structural changes include cardiomyocyte hypertrophy and extensive interstitial fibrosis [46]. Fibrosis may disrupt myocardial electrical continuity by interfering with inter-myocyte coupling, promoting AF [12,47]. Known mediators that promote fibrosis include angiotensin II (AngII) [48], transforming growth factor beta 1 (TGF-β1) [49] and platelet-derived growth factor (PDGF) [50]. Furthermore, matrix metalloproteinase has been implicated in the remodeling of the extracellular matrix (ECM) of the heart [46,51]. Additionally, structural remodeling is mutually associated with AF: not only do structural remodeling events, such as fibrosis, promote AF, but AF itself also promotes cardiac fibrosis [52].

## 3. MicroRNAs (miRNAs)

MicroRNAs are a class of short (approximately 21–23 nts long), non-coding RNAs that bind to target mRNAs and participate in either translation repression or degradation, functioning as important post-transcriptional gene regulators [53]. In the genome, miRNAs are encoded as either intronic or intergenic miRNAs [54]. In the former case, miRNAs are processed from the introns of protein-coding gene transcripts, whereas they are transcribed under the control of their own promoters in the latter case. miRNA biogenesis begins with the transcription of a primary transcript, pri-miRNA, by RNA polymerase II in the nucleus. Pri-miRNAs, mRNA molecules that are generally thousands of nts long, are then processed by the ribonuclease III, Drosha, to produce a premature miRNA molecule (pre-miRNA) that is approximately 100 nt long and has a hairpin-like structure. The pre-miRNA is subsequently transferred to the cytosol by the nuclear export factor, exportin 5; the ribonuclease III, Dicer, processes this pre-miRNA further to generate a mature miRNA (for a detailed review of miRNA biogenesis, see [55]). Since they were first discovered in 1993 [56], miRNAs have been implicated in various diseases, including cardiovascular diseases [57,58,59]. According to a recent review (published in January 2014) regarding the role of miRNAs in AF, less than 10 miRNAs (miR-1, -21, -26, -29, -30, -133, -328 and -499) have been empirically associated with the regulation of the cardiac remodeling process [3]. However, our recent literature search for this particular review came up with additional miRNAs that may be involved in AF development; these miRNAs will be discussed in the corresponding section to come.

Regarding the influential range of miRNAs, recent studies have indicated that miRNAs might also exist as extracellular miRNAs. The known types of extracellular miRNAs are believed to be: (1) vesicle free; (2) apoptotic body enclosed; (3) vesicle shedding and exosome packaged; and (4) LDL associated [60]. Among these forms, the vesicle-free types, the apoptotic body-enclosed types and the exosome-packaged types are considered to be more relevant to oxidative injury/cell death situations. The apoptotic body-enclosed types are generated during apoptosis, as the name suggests. However, experimental data have suggested that the miRNAs contained in apoptotic bodies could function as an anti-inflammatory mechanism, rather than as a source of inflammation [61]. In contrast, the vesicle-free types have been suggested to be released passively by cell lysis in the form of complexes with Ago proteins during cell death [62], and for the exosome-packaged types, their release has been reported to be enhanced by oxidative stress [63]. It was demonstrated that miRNAs could bind to single-stranded, RNA-sensing Toll-like receptors (TLRs), such as TLR8 [64]. Because TLRs are important mediators of innate immunity [65], extracellular miRNA-mediated local inflammatory response and subsequent damage to surrounding tissue can occur. In fact, extracellular miRNAs, such as let-7, miR-21 and miR-29a, have been reported to induce an inflammatory response by binding to TLRs [66,67]. These data suggest that the range that is influenced by ROS-mediated regulation of miRNAs might not be limited to the cells directly exposed to ROS, and the impact of ROS-mediated regulation of miRNA might be greater than it appears.

## 4. Reactive Oxygen Species (ROS)

Oxidative stress has been implicated in the development and progression of various human diseases, including cardiovascular diseases, and is associated with excess ROS production [68,69]. The heart consumes a large amount of oxygen to maintain essential cellular functions and aerobic metabolism, during which both adenosine triphosphate (ATP) and ROS are generated in mitochondria [70]. ROS include a number of highly-reactive, free, non-radical and partially-reduced oxygen metabolites, such as the superoxide anion radical (·O_2_^−^), nitric oxide (NO), peroxynitrite (ONOO^−^) and hydrogen peroxide (H_2_O_2_) [71].

ROS participate in several important cellular signaling pathways as second messengers of growth factors and cytokines and also regulate gene expression by modulating transcription factors [72,73]. The increases in ROS are caused by an imbalance between ROS-producing enzymes (*i.e.*, nicotinamide adenine dinucleotide phosphate, or NAD[P]H-oxidase, xanthine oxidase, the mitochondrial electron-transport chain and dysfunctional uncoupled eNOS) and antioxidant enzymes (*i.e.*, superoxide dismutase (SOD), catalase, glutathione peroxidase, heme oxygenase and paraoxonase) [74]. The high rate of ROS production causes cell damage and death by impairing DNA, protein, cell membrane and cellular organelle functions, leading to the development of cardiovascular disease [75,76]. In the case of AF, various ROS molecules target arrhythmogenic molecules, including CaMKII [77], RyR2 [78] and LTCC [79] (for a more comprehensive review of the role of ROS in AF, see [80]).

ROS, such as superoxide and H_2_O_2_, are also known to modulate the generation of nitrogen oxide (NO) by regulating nitric oxide synthase (NOS). For example, H_2_O_2_ either increases or decreases endothelial NOS activity, depending on its concentration [81,82,83]. Furthermore, superoxide reacts with NO to produce peroxynitrite [84]. Peroxynitrite can induce the uncoupling of NOS, thus decreasing the bioavailability of NO [85], which can impair normal heart function [86]. Because the important role of NO in the development of AF has long been recognized [87,88], the redox balance achieved by these ROS is expected to have a significant impact on the development of AF.

Additionally, in a study using isolated animal hearts, the causal role of oxidative stress in AF was demonstrated [89]. However, in humans, it is virtually impossible to conduct an experiment that could directly show the cause and effect relationship between ROS and AF. Consequently, most of the available human data indicating the involvement of ROS in the development of AF are analyses of clinical, observational outcomes in patients (*i.e.*, correlations between oxidative stress markers in AF patients [90] or pro- and anti-oxidant gene expression analyses in AF patients [91]). Additionally, less than satisfactory clinical results of ROS scavengers in treating AF [92] indicated that ROS might not be the single most important factor in the development of AF in humans. Thus, if we had to summarize, based on our current understanding on the subject, we would assert the following: although there is ample empirical evidence (of mostly animal studies) identifying ROS as the cause of AF, because AF in humans is a complex disease with a multifactorial etiology, it cannot be simply stated that ROS is definitively the most important factor responsible for the development of AF in humans.

## 5. ROS, miRNA and AF

Accumulating evidence indicates that miRNAs play an essential role in arrhythmogenesis [93]. To catch up with recent advances in miRNA-mediated arrhythmogenesis, we conducted a literature search in PubMed, using miRNA and arrhythmia as key words. Among the 130 articles we found (as of 10 October 2014), reviews and articles with irrelevant contents were excluded, as only original articles with relevant content were selected for this particular review. Key miRNAs that have been implicated or may be potentially involved in arrhythmogenesis are summarized in Table 1. Individual miRNAs implicated in AF pathogenesis by modulating electrical and structural remodeling processes are discussed in detail below.

**Table 1:** miRNAs with reported or possible associations with atrial fibrillation (AF).

miRNA	Changes in CVDs (Ref. if miR Expression Was Confirmed in Other Study)	Targeted Protein (mRNA) and Subsequent Results		Ref.
**miR-1**	Decreased in AF	Potassium channel, inwardly rectifying, Kir2.1 (KCNJ2)	Increased *I*_k1_	[94]
	Decreased in hypertrophy	Connexin 43 (GJA1)	Cx43 displacement due to hyper-phosphorylation	[95]
	Increased in HF ([96])	Protein phosphatase 2A (PPP2CA)	Excessive RyR2 phosphorylation by CaMKII	[97]
**miR-19a**	Increased in AF ([98])	Phosphatase and tensin homolog (PTEN)	Increased hypertrophy	[99]
		Connexin 43 (GJA1)	Disturbed electrical coupling	
**miR-21**	Increased in MI Increased in AF	Sprouty1 (SPRY1)	Increased fibrosis	[100]
		Calcium channel, voltage-dependent, l type, alpha 1C subunit (CACNA1C)	Shortened APD	[101]
		Calcium channel, voltage-dependent, beta 2 subunit (CACNB2)	Decreased *I*_CaL_	
**mir-26**	Decreased in AF	Potassium channel, inwardly rectifying, Kir2.1 (KCNJ2)	Increased *I*_k1_	[102]
**miR-29b**	Decreased in CHF	Collagen, type I, alpha 1 (COL1A1)	Increased fibrosis	[103]
		Collagen, type III, alpha 1 (COL3A1)		
		Fibrillin (FBN)		
**miR-30**	Reduced in AF ([104])	Connective tissue growth factor (CTGF)	Increased fibrosis	[105]
**mir-130a**	Increased in Atherosclerosis ([106])	Connexin 43 (GJA1)	Disturbed electrical coupling	[107]
**miR-133**	Reduced in AF ([104])	Connective tissue growth factor (CTGF)	Increased fibrosis	[105]
	Increased in HF ([96])	Protein phosphatase 2A (PPP2CA)	Excessive RyR2 phosphorylation by CaMKII	[97]
**miR-145**	Decreased in AF ([108])	Ca2+/calmodulin-dependent protein kinase 2 delta (CAMK2D)	Excessive RyR2 phosphorylation by CaMKII	[109]
**miR-328**	Increased in AF	Calcium channel, voltage-dependent, l-Type, alpha 1C subunit (CACNA1C)	Shortened APD	[108]
	Increased in hypertrophy ([ 110])	Calcium channel, voltage-dependent, beta 1 subunit (CACNB1)	Decreased *I*_CaL_	[111]
**miR-499**	Increased in AF	Small-conductance calcium-activated potassium channel 3 (KCNN3)	Altered conduction	[112]
	Increased in hypertrophy ([113])		Increased AF	
**Let-7e**	Decreased in Acute MI	Beta 1 adrenergic receptor (ADRB1)	Increased arrhythmia score	[114]

AF, atrial fibrillation; HF, heart failure; MI, myocardial infarction; CHF, congestive heart failure; APD, action potential duration.

### 5.1. miRNAs that Likely Contribute to Arrhythmogenesis

#### 5.1.1. miR-1

Among the conduction- and membrane potential-related ion channels of cardiomyocytes, *GJA1* (encodes connexin 43, Cx43) and *KCNJ2* (encodes K^+^ channel subunit Kir2.1) are targeted by miR-1. miR-1 expression is reportedly decreased in AF, allowing for increased I_K1_ activity, which is mediated by Kir2.1 [94]. Decreased miR-1 expression has also been reported in cardiac hypertrophy, a decrease that resulted in tachyarrhythmia due to the hyper-phosphorylation of Cx43 and subsequent Cx43 displacement [95]. These studies demonstrated the importance of miR-1 regulation in the electrical remodeling of the heart.

Another possible mechanism of miR-1-mediated arrhythmogenesis was proposed by Teremtuev *et al*. In their over-expression study, increased miR-1 levels in cardiac myocytes resulted in selective decreases in the expression of the B56α regulatory subunit of protein phosphatase 2A (PP2A), which mediates LTCC and RyR2 dephosphorylation. This decrease consequently increased the phosphorylation of LTCC and RyR2 in a CaMKII-dependent manner and resulted in significant increases in inward Ca^2+^ current (*I*_Ca_) and Ca^2+^ release from the SR [97]. In fact, such an miR-1-mediated arrhythmogenic mechanism was confirmed in a heart failure model a few years later [96]. As both a decrease and an increase in miR-1 contributed to arrhythmogenesis, the findings of this study may seem contradictory. However, they may also indicate that the underlying pathologic conditions of the heart that lead to arrhythmogenesis can vary and that different conditions employ different mechanisms of utilizing miRNA; therefore, even a single miRNA may be regulated differently in the setting of different pathological conditions of the heart.

#### 5.1.2. miR-19a (miR-17-92 Cluster)

The miR-17-92 cluster consists of the following six mature miRNAs: miR-17, -18a, -19a, -19b, -20a and -92a [115]. The roles of miR-17-92 in cardiovascular diseases, including arrhythmias, have been not well elucidated compared with cancer pathogenesis [116]. The expression of miR-19a, one of the members of the miR-17-92 cluster, is reportedly increased in the plasma of patients with AF [98]. Furthermore, when over-expressed in mice, the miR-17-92 cluster, including miR-19a/b, repressed the expression of PTEN, which regulates cardiomyocyte structure, size and contractility [117], and Cx43, which eventually resulted in premature sudden death [99]. Such results suggested that the miR-17-92 cluster was involved in both electrical and structural remodeling processes.

#### 5.1.3. miR-21

In a rat model of myocardial infarction (MI), the level of atrial miR-21 expression increased, which was accompanied by increased left ventricular enlargement, hypo-contractility, left atrial dilation, fibrosis, refractory period prolongation and AF [100]. In that particular study, miR-21 promoted fibrotic remodeling following myocardial infarction by down-regulating Sprouty-1 (Spry1), a protein known to suppress fibroblast proliferation [118], to negatively regulate extracellular signal-regulated kinases 1/2 (ERK1/2) [119] and to up-regulate collagen-1 and 3 expression. According to a recent study, increased miR-21 expression was believed to result in the down-regulation of dual-specificity phosphatase 8 (DUSP8), a negative regulator of c-Jun *N*-terminal kinase (JNK) and p38 mitogen-activated protein kinase (p38) [120], leading to the promotion of collagen synthesis in cardiac fibroblasts [121]. In another study, in the cardiomyocytes of patients with chronic AF, miR-21 expression increased, which resulted in the subsequent down-regulation of two subunits of a voltage-dependent calcium channel, specifically subunits 1αC (CACNA1C) and β2 (CACNB2), resulting in reduced l-Type Ca^2+^ current (*I*_CaL_) [101]. As reduced *I*_CaL_ activity is one of the hallmarks of AF [15], this study demonstrated that miR-21 can be pro-arrhythmogenic and can modulate the electrical remodeling of cardiomyocytes.

#### 5.1.4. miR-26

It has been reported that miR-26 also targets Kir2.1 and is down-regulated in humans and animals with AF. The decrease in miR-26a expression induced I_K1_ dysregulation via Kir2.1 expression, which is related to AF susceptibility. Interestingly, the AF-induced miR-26 decrease was mediated by a Ca^2+^-dependent transcription factor, nuclear factor of activated T-cells (NFAT) [102]. As increased *I*_K1_ activity is commonly observed in AF [27,28], miR-26 is believed to contribute to AF development by participating in the electrical remodeling of cardiomyocytes.

#### 5.1.5. miR-29b

Dawson *et al.* reported that miR-29b expression decreased rapidly in the left atrial tissues studied in a congestive heart failure (CHF)-related AF model involving canines. [103]. The major targets of miR-29b identified in that study were ECM-coding genes, such as *COL1A1*, *COL3A1* and *FBN* (encodes fibrillin); these genes were up-regulated in CHF fibroblasts due to decreased miR-29b expression. The expression of this miRNA is believed to be controlled by TGF-β-mediated NF-κB activation, because TGF-β expression is up-regulated in the arrhythmogenic atria of both animals and humans [122,123]. These studies indicate that miR-29 is involved in AF-related structural remodeling.

#### 5.1.6. miR-30

Connective tissue growth factor (CTGF) is an important molecule in fibrosis progression, and its expression is reportedly regulated by two miRNAs, miR-30 and -133 [105]. In that study, both miR-30 and -133 expression levels were decreased in pathological left ventricular hypertrophy, which led to the promotion of collagen synthesis via CTGF up-regulation. Down-regulation of miR-30 and -133 in AF was further confirmed in another study by Li *et al.* [104]. These results indicate that both miR-30 and -133 modulate structural remodeling in AF by regulating fibrotic proteins.

#### 5.1.7. miR-130a

Along with miR-27b and -210, -130a is reportedly increased in the serum in atherosclerosis obliterans [106]. Cardiac-specific overexpression of miR-130a in transgenic (Tg) mice resulted in decreased fractional shortening and in irregular heart rhythms, which were noted via echocardiographic analysis; compared with normal mice, miR-130a Tg mice demonstrated the following typical characteristics of atrial tachyarrhythmia: an irregular ventricular rate and rapid arterial activity in simultaneous surface and intracardiac electrocardiograms. These negative effects of ectopic miR-130a expression were linked to reduced Cx43 protein expression [107]. This study demonstrated that by modulating the expression of Cx43, miR-130a contributes to both electrical remodeling and the development of cardiac arrhythmias.

#### 5.1.8. miR-133

In addition to contributing to the fibrotic structural remodeling of cardiomyocytes by targeting CTGF (see Section 5.1.6. above), miR-133 also targets the B56α regulatory subunit, PP2A, as does miR-1 (see Section 5.1.1. above). Resultant increases in LTCC and RyR2 phosphorylation caused significant increases in *I*_Ca_ activity and Ca^2+^ release from the SR in a heart failure model [96]. These studies suggest that miR-133 is involved in both the structural and the electrical remodeling of cardiomyocytes in the setting of arrhythmogenesis, as is the case with miR-19a.

#### 5.1.9. miR-145

CaMKIIδ is a target [109] of miR-145, the expression of which is reportedly decreased in AF [108]. As increased CaMKII activity and subsequent increases in RyR2 phosphorylation resulted in arrhythmogenic SR Ca^2+^ leakage [124], the increase in CaMKII expression caused by the down-regulation of miR-145 in AF is also believed to be pro-arrhythmogenic. Therefore, miR-145 is believed to be involved in the electrical remodeling of cardiomyocytes in the setting of AF.

#### 5.1.10. miR-328

miR-328 has been linked to cardiac hypertrophy, although its underlying mechanisms of action remain elusive. In a previous study in which miR-328 over-expression was accompanied by a reduced SERCA2a level, an increased intracellular calcium concentration and an increased calcineurin protein level, the importance of miR-328 in cellular calcium handling was demonstrated [110]. Another study conducted on animals and patients with AF reported that miR-328 is aberrantly up-regulated and targets CACNA1C and CACNB1 in AF, which results in reduced *I*_CaL_ activity and a shortage of APD [111]. Additionally, circulating levels of miR-328 were associated with increases in AF prevalence [125], suggesting the potential involvement of miR-328 in the electrical remodeling and calcium handling processes of AF.

#### 5.1.11. miR-499

miR-499 is reportedly up-regulated in both human and murine cardiac hypertrophy and cardiopathy, is sufficient to cause heart failure in murine subjects and accelerates maladaptation to pressure overloading [113]. Regarding the role of miR-499 in AF, Ling *et al.* reported that miR-499 was increased in AF and targeted the small-conductance calcium-activated potassium channel 3 (SK3, encoded by KCNN3 [126]), contributing to the electrical remodeling characteristics of AF [112].

#### 5.1.12. Let-7e

Beta-adrenergic receptors (β-ARs) are G-protein-coupled receptors and play important roles in the regulation of cardiac function and heart rate [127]. The involvement of β-ARs in AF development has been indirectly demonstrated by the effectiveness of β-blockers at preventing AF [128]. According to a paper published by Li *et al.*, in a rat model of acute MI, β_1_-AR expression increased, whereas cardiac-enriched let-7 family miRNA expression levels decreased, indicating that β_1_-AR is a direct target of let-7e [114]. In the same study, β_1_-AR down-regulation by exogenous let-7e exerted an anti-arrhythmic effect in MI rats, suggesting that let-7e may represent a novel therapeutic target for ischemia-induced cardiac arrhythmia by modulating β_1_-AR expression.

### 5.2. AF-Related miRNAs and ROS

For this section, we conducted a literature review to find any reported or possible connection between the aforementioned AF-related miRNAs and ROS to determine whether it is possible that those miRNAs are modulated by ROS and that ROS-induced arrhythmogenesis is at least partially mediated by miRNAs. For the literature search, key words, such as ROS, hydrogen peroxide (or H_2_O_2_, the compound most commonly used to simulate ROS *in vitro*) and oxidative stress, were used, along with individual miRNAs. Known or plausible relationships between the AF-related miRNAs and ROS are summarized in Table 2.

**Table 2:** Modulation of AF-related miRNAs by ROS.

miRNA	Modulation by ROS	Stimulation	Experimental System	Ref.
miR-1	Increased	H_2_O_2_ (50–400 µM)	H9c2	[129]
	Increased	H_2_O_2_ (100 µM, 6 h)	NRCM	[130]
	Decreased	H_2_O_2_ (100 µM, 6 h)	NRVM	[131]
miR-19a	Decreased	H_2_O_2_ (200 µM, 6 h)	VSMC	[132]
miR-21	Increased	H_2_O_2_ (10–100 µM, 6 h)	NRCM	[133]
	Increased	H_2_O_2_ (25–200 µM, 6 h)	VSMC	[132]
	Increased	H_2_O_2_ (100 µM, 6 h)	NRVM	[131]
miR-26	Increased	H_2_O_2_ (100 µM, 6 h)	NRCM	[130]
	Decreased	H_2_O_2_ (200 µM, 6 h)	VSMC	[132]
miR-29b	Decreased	H_2_O_2_ (200 µM, 6 h)	VSMC	[132]
miR-30	Decreased	H_2_O_2_ (100 µM, 6 h)	NRCM	[134]
miR-130a	Decreased	H_2_O_2_ (200 µM, 6 h)	VSMC	[132]
miR-133	Decreased	H_2_O_2_ (100 µM, 24 h)	NRCM	[135]
	Decreased	H_2_O_2_ (100 µM, 6 h)	NRVM	[131]
miR-145	Decreased	H_2_O_2_ (50 µM, 0.5–8 h)	NRVM	[136]
miR-328	Decreased	H_2_O_2_ (200 µM, 6 h)	VSMC	[132]
miR-499	Increased	H_2_O_2_ (50–200 µM, 6 h)	NRVM	[131]
Let-7e	Decreased	H_2_O_2_ (50 µM, 1 h)	HCT116 colon cancer cells	[137]
	Increased	H_2_O_2_ (200 µM, 6 h)	VSMC	[132]

NRCM, neonatal rat cardiomyocytes; VSMC, vascular smooth muscle cells; NRVM, neonatal rat ventricular myocytes.

**Figure 1:**
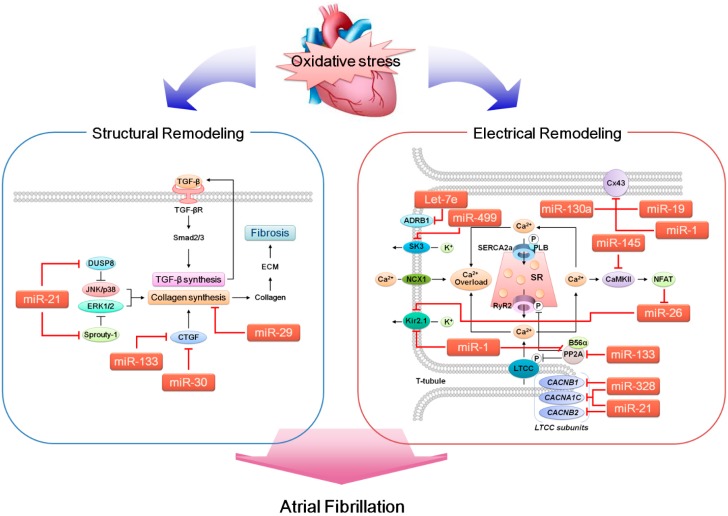
Concept of the ROS–miRNA–AF framework.

As shown in Table 2, in terms of expression levels in cardiomyocytes, more than half of the AF-related miRNAs (seven out of 12 miRNAs) were affected by the presence of ROS (H_2_O_2_). Additionally, the same number of miRNAs (seven out of 12 miRNAs, as well) reportedly changed their expression levels (*i.e.*, were either up- or down-regulated) in response to H_2_O_2_ treatment in vascular smooth muscle cells (VSMCs). VSMCs are an important vascular cell line and contribute significantly to the pathogenesis of various cardiovascular diseases [138]. Therefore, given that a positive association between AF and vascular diseases, such as atherosclerosis, has been clinically established in population studies [139,140], it is not far-fetched to assume that even the ROS-dependent regulation of those AF-related miRNAs in VSMCs may predispose individuals to AF development. Because changes in the expression levels of a particular miRNA varied depending on the experimental conditions (*i.e.*, cell types or H_2_O_2_ concentrations), it is difficult for this review to provide any generalized or conclusive evidence regarding the existence of a framework composed of ROS–miRNA–AF. However, given that key electrical remodeling-related molecules, such as CaMKII, RyR, LTCC, SERCA and NCX, are targeted by ROS [80] and that an entire array of miRNAs with the potential to affect the arrhythmogenic process (*i.e.*, targeting ion channels [108]) has not been investigated regarding any possible ROS-dependent regulation, the odds of the existence of such an ROS–miRNA–AF framework in the setting of arrhythmogenesis are reasonably high (Figure 1).

### 5.3. Perspective: Can ROS-Dependent Dysregulation of miRNA Really Cause AF in Humans?

In studies using animals, excessive ROS (*i.e.*, 10–200 µM of H_2_O_2_; see Table 2) are used to simulate oxidative stress, which occurs when the amount of ROS exceeds the cell’s ability to cope with it, thus proving the concept. However, we do not believe that endogenous ROS reach such high levels frequently [141,142], and more importantly, the etiology of AF in humans is more complicated than simply being driven by a single factor, such as ROS. In fact, other factors, such as inflammation and myocardial fibrosis, are also known to promote AF in humans [143,144]. Thus, in our opinion, endogenous oxidative stress most likely acts both as one of the AF triggering factors and as a factor exacerbating AF. Consequently, we believe that most people do not develop AF, because the initiation of AF in humans requires more than just high levels of ROS, and pathological manifestations occur only when other pathological conditions are “unfortunately satisfied”.

Nevertheless, there is a possibility that one might be more susceptible to AF than another, depending on the genetic composition of the individual. In fact, a correlation between an SNP (single nucleotide polymorphism) and AF was reported [145]. Additionally, individual personal lifestyles can have effects on the probability of developing AF; smoking [146] and drinking [147] are good examples. Another theoretically possible case involving miRNAs is one having SNPs on the coding sequences of miRNAs involved in the regulation of AF development. Similar to SNPs on protein coding genes, SNPs on miRNA coding sequences can result in the demise of miRNA function [148]. Thus, in theory, SNP-induced dysregulation of miRNA in an individual can increase (or decrease) his or her susceptibility to AF, and a very recent study reported such a possibility [149].

## 6. Concluding Remarks

It should be noted that this review was mostly based on the results of experimental studies on animals. Because AF in humans is a complex disease with a multifactorial etiology (*i.e.*, oxidative stress, inflammation and atherothromboembolism [150]), the outcomes of animal studies might not perfectly reflect the actual etiology of AF in humans, which is an obvious limitation of this review, as well as of most studies involving animal subjects in general, and readers should consider this point in interpreting this review. Another limitation of this review is that the number of publications regarding the ROS-dependent regulation of AF-related miRNAs was very limited; therefore, this review covered only the possible ROS-dependent regulation of those AF-related miRNAs discussed in earlier sections of this manuscript. However, the possibility that ROS affects miRNAs and that such ROS-dependent changes to miRNAs either directly or indirectly contribute to arrhythmogenesis still exists. Therefore, the aim of this review was to encourage scientific interest in discovering a conceptual framework composed of ROS–miRNA–AF that is related to arrhythmia development. Although this review could not provide hard evidence of any specific, detailed examples of such a framework, the fact that previously-described AF-related miRNAs changed in response to ROS was enough to suggest the possibility that miRNAs translate cellular stressors, such as ROS, into AF pathogenesis and warrants further study with a more appropriate experimental design to prove or disprove the existence of an ROS–miRNA–AF framework.
